# *Eubacterium rectale* Improves the Efficacy of Anti-PD1 Immunotherapy in Melanoma via l-Serine-Mediated NK Cell Activation

**DOI:** 10.34133/research.0127

**Published:** 2023-04-28

**Authors:** Nian Liu, Lihui Chen, Mingjie Yan, Qian Tao, Jie Wu, Jing Chen, Xiang Chen, Wei Zhang, Cong Peng

**Affiliations:** ^1^ Department of Clinical Pharmacology, Xiangya Hospital, Central South University, Changsha, Hunan, China.; ^2^ Department of Dermatology, Xiangya Hospital, Central South University, Changsha, Hunan, China.; ^3^ Hunan Key Laboratory of Skin Cancer and Psoriasis, Xiangya Hospital, Central South University, Changsha, Hunan, China.; ^4^ Furong Laboratory, Xiangya Hospital, Central South University, Changsha, Hunan, China.; ^5^ National Clinical Research Center for Geriatric Disorders, Xiangya Hospital, Central South University, Changsha, Hunan, China.

## Abstract

Natural killer (NK) cells, as key immune cells, play essential roles in tumor cell immune escape and immunotherapy. Accumulating evidence has demonstrated that the gut microbiota community affects the efficacy of anti-PD1 immunotherapy and that remodeling the gut microbiota is a promising strategy to enhance anti-PD1 immunotherapy responsiveness in advanced melanoma patients; however, the details of the mechanism remain elusive. In this study, we found that *Eubacterium rectale* was significantly enriched in melanoma patients who responded to anti-PD1 immunotherapy and that a high *E. rectale* abundance was related to longer survival in melanoma patients. Furthermore, administration of *E. rectale* remarkably improved the efficacy of anti-PD1 therapy and increased the overall survival of tumor-bearing mice; moreover, application of *E. rectale* led to a significant accumulation of NK cells in the tumor microenvironment. Interestingly, conditioned medium isolated from an *E. rectale* culture system dramatically enhanced NK cell function. Gas chromatography–mass spectrometry/ultrahigh performance liquid chromatography–tandem mass spectrometry-based metabolomic analysis showed that l-serine production was significantly decreased in the *E. rectale* group; moreover, administration of an l-serine synthesis inhibitor dramatically increased NK cell activation, which enhanced anti-PD1 immunotherapy effects. Mechanistically, supplementation with l-serine or application of an l-serine synthesis inhibitor affected NK cell activation through Fos/Fosl. In summary, our findings reveal the role of bacteria-modulated serine metabolic signaling in NK cell activation and provide a novel therapeutic strategy to improve the efficacy of anti-PD1 immunotherapy in melanoma.

## Introduction

Melanoma is a lethal and treatment-resistant skin tumor [1]. Recently, immune checkpoint inhibitor (ICI) therapy was shown to significantly prolong the survival of advanced melanoma patients [2]. However, at most, 30% of patients show a clinical response to ICIs, and 60 to 70% of patients develop primary or acquired resistance [3]. Therefore, the identification of responsive biomarkers and the development of combination therapeutic approaches to improve the efficacy of ICIs are urgently needed for the clinical treatment of melanoma.

The role of the gut microbiome in the regulation of innate immunity as well as adaptive immunity has been well documented [4]. Innate immunity acts as the first line of defense against pathogenic microorganism invasion, initiating a primary immune response to block pathogen dissemination through the expression of inflammatory factors, including related cytokines and chemokines, such as CCL2, interleukin-13 (IL-13), and IL-21 [5–7], and facilitating T cell or B cell activation to generate local or whole-body immune responses. Regarding adaptive immune responses, intestinal immune homeostasis plays a critical role in immune regulation to avoid host immune dysfunction [8].

Recently, growing evidence has revealed that the gut microbiota also modulates the efficacy of tumor therapy, particularly immunotherapy [9]. Fecal microbiota transplantation (FMT) results demonstrated that mice transplanted with stool derived from PD1 blockade responders showed stronger antitumor activity after anti-PD1 treatment than FMT mice transplanted with stool from nonresponders [9]. Furthermore, clinical trials have demonstrated that FMT is a potentially promising strategy to enhance ICI efficacy, as this approach can overcome primary or acquired resistance in various tumors, including melanoma [10–13]. In addition, accumulating evidence has revealed that specific microbial species influence ICI efficacy or act as prognostic markers for ICI therapy. For example, administration of *Akkermansia muciniphila* significantly improved the efficacy of anti-PD1 therapy in melanoma by triggering dendritic cell (DC) activation [14], suggesting that supplementation with probiotics might be another combination strategy for enhancing the efficacy of ICI.

Here, using a public macrogenomic database, we identified *E. rectale* that was significantly enriched in the intestinal flora of patients who responded to immune checkpoint therapy, particularly those who responded to anti-PD1 immunotherapy. *E. rectale*, a Gram-positive bacterium, belongs to the family *Lachnospiraceae (f_Lachnospiraceae)* and genus *Lachnospiraceae_unclassified (g_Lachnospiraceae _unclassified)*. Melanoma patients with a high abundance of *E. rectale* had longer survival. *E. rectale* makes up 13% of total human colonic feces, is one of the most prevalent bacterial species, and is important for the maintenance of human health [15–17]. In Behçet's disease, *E. rectale* reduces pathogenesis by regulating DCs [18]. In addition, *E. rectale* plays a key role in the development of tumors. In a study of patients with pancreatic ductal adenocarcinoma (PDAC), *E. rectale* abundance was found to be significantly reduced in the gut microbiota of the patients, leading to dysregulation of the intestinal barrier and thus promoting the development of pancreatic cancer. Moreover, the authors found that *E. rectale* could be used as a biomarker to distinguish PDAC patients from healthy controls [19]. The *E. rectale* abundance was significantly reduced in the gut microbiota of patients with lymphoma. Supplementation with *E. rectale* reduced tumor necrosis factor levels and the incidence of lymphoma in sensitized Eμ-Myc mice [20]. However, the details of the relevance of *ER* in melanoma have not been fully elucidated.

In our study, we confirmed the sensitizing effect of *E. rectale* for anti-PD1 immunotherapy in a preclinical model and found that *E. rectale* enhanced the potentiating effect of natural killer (NK) cells on anti-PD1 immunotherapeutic efficacy by depleting l-serine in the environment.

## Results

### *E. rectale* abundance was positively correlated with anti-PD1 therapeutic effects and patient survival

To precisely identify bacterial species that improve the prognosis of melanoma patients receiving ICI immunotherapy, we included 2 studies correlating the gut microbial composition with the efficacy of ICI therapy in a melanoma database and analyzed the raw macrogenome (metagenome shotgun sequencing) data of cohort 1 [21]. The results showed that 4 enriched species in the treatment-responsive (R) group and 5 enriched species in the treatment-nonresponsive (NR) group influenced the clinical response to anti-PD1/anti-CTLA4 treatment by linear discriminant analysis effect size (LEfSe) analysis [linear discriminant analysis (LDA) ≥ 3; *P* < 0.05] (Fig. S1A and B). The α-diversity and β-diversity were not significantly different between the R and NR groups (Fig. S1C and D). Additionally, we compared the abundance of the 9 differentially abundant species (*P* < 0.05, Wilcoxon test) and found that the abundance of *E. rectale* in the R group was markedly increased (Fig. S1E). Moreover, when considering the 4 R-enriched species, patients with a higher *E. rectale* abundance had longer progression-free survival (PFS) [median PFS (mPFS): 5.7 months versus 2.8 months, *P* = 0.036] (Fig. S1F), whereas the abundance of the other differentially enriched species was not associated with patient survival (Fig. S1G to I), indicating that *E. rectale* might play a critical role in ICI therapy.

**Fig. 1.**
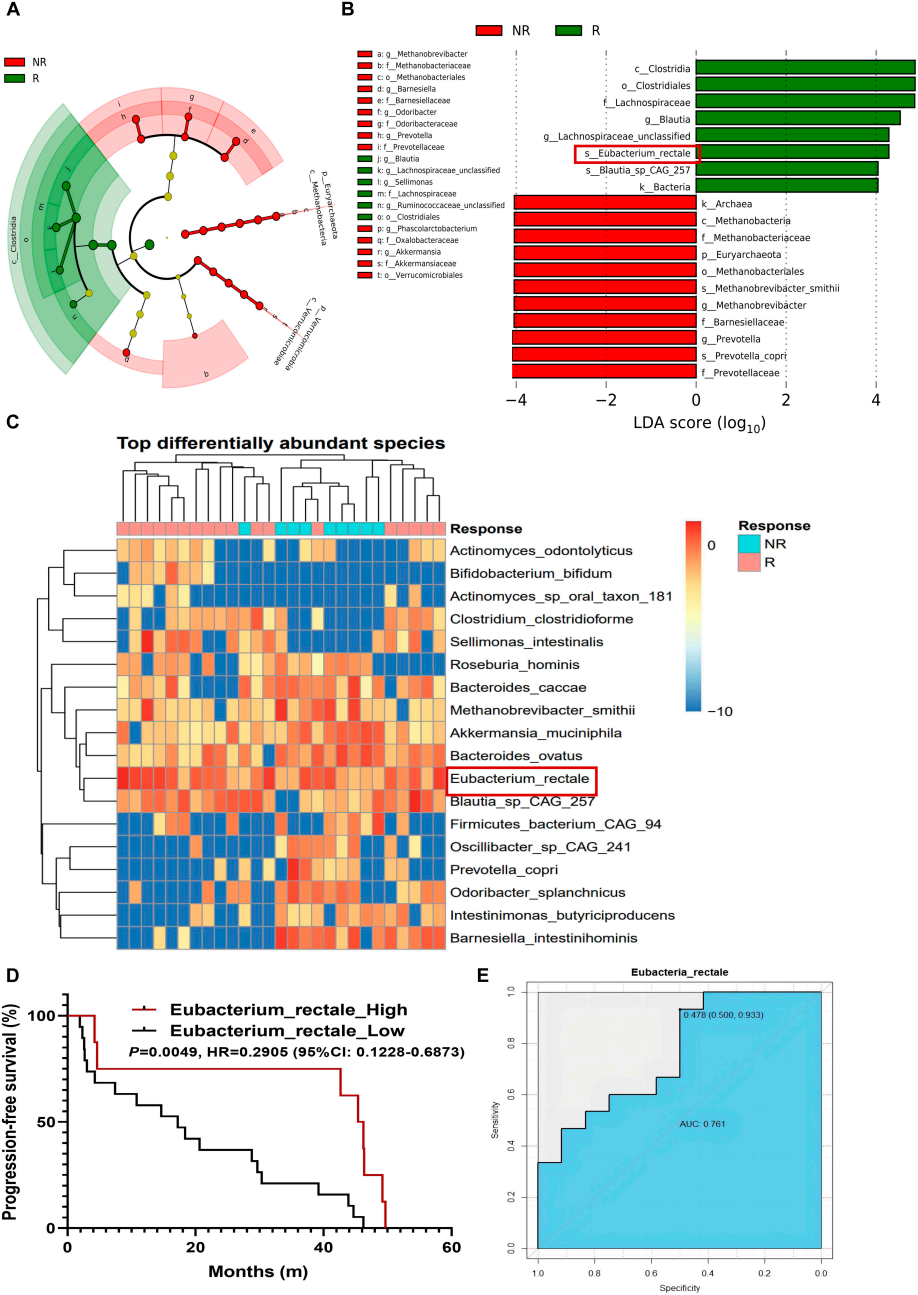
Abundance of *E. rectale* predicts patient responsiveness to anti-PD1 therapy. (A and B) Results of LEfSe analysis for the R and NR groups. (A) Taxonomic cladogram from LEfSe showed different taxa enriched in the R and NR group (LDA ≥ 4; *P* < 0.05). (B) LEfSe identified significantly differentially abundant taxa in the R and NR groups (LDA ≥ 4; *P* < 0.05). (C) Heatmap of relative abundance differences between the R and NR groups at the species level (Wilcoxon test). (D) The Kaplan–Meier method with log-rank test estimates the mPFS for patients with higher or lower abundance of *E. rectale*. (E) A prediction model was constructed by *E. rectale* in the training set, and predictive performance on the testing set was evaluated by ROC curves. R (*n* = 14), NR (*n* = 11). Multiple experimental data were counted and are presented according to the statistical method, and an asterisk (*) indicates the *P* value.

To further verify the above results, we selected another cohort of anti-PD1-treated patients for analysis (cohort 2) [22] and found that *E. rectale* was the most differential species between the R and NR groups (LDA ≥ 4; *P* < 0.05) (Fig. 1A and B). As expected, *E. rectale* was significantly enriched in the intestinal flora of melanoma patients in the responding group, as shown in Fig. 1C. Similarly, patients with a higher *E. rectale* abundance had longer PFS in cohort 2 (Fig. 1D). In addition, we used cohort 1 as the training set and cohort 2 as the validation set and generated separate prediction models using the abundance of *E. rectale* obtained from cohort 1. As shown in Fig. 1E, we evaluated the performance of the model using the area under the curve (AUC) of the receiver operating characteristic (ROC) and showed that the abundance of *E. rectale* predicted clinical response in patients in cohort 2 (AUC = 0.761) (Fig. 1E). Based on the results of the analysis of the 2 cohorts, we revealed that *E. rectale* was associated with survival and patient responsiveness to anti-PD1 therapy; these results suggested that *E. rectale* might serve as a biomarker to predict anti-PD1 treatment responsiveness and exert an important function in anti-PD1 therapy in melanoma.

### *E. rectale* improved the efficacy of anti-PD1 immunotherapy through NK cells in melanoma

**Fig. 2.**
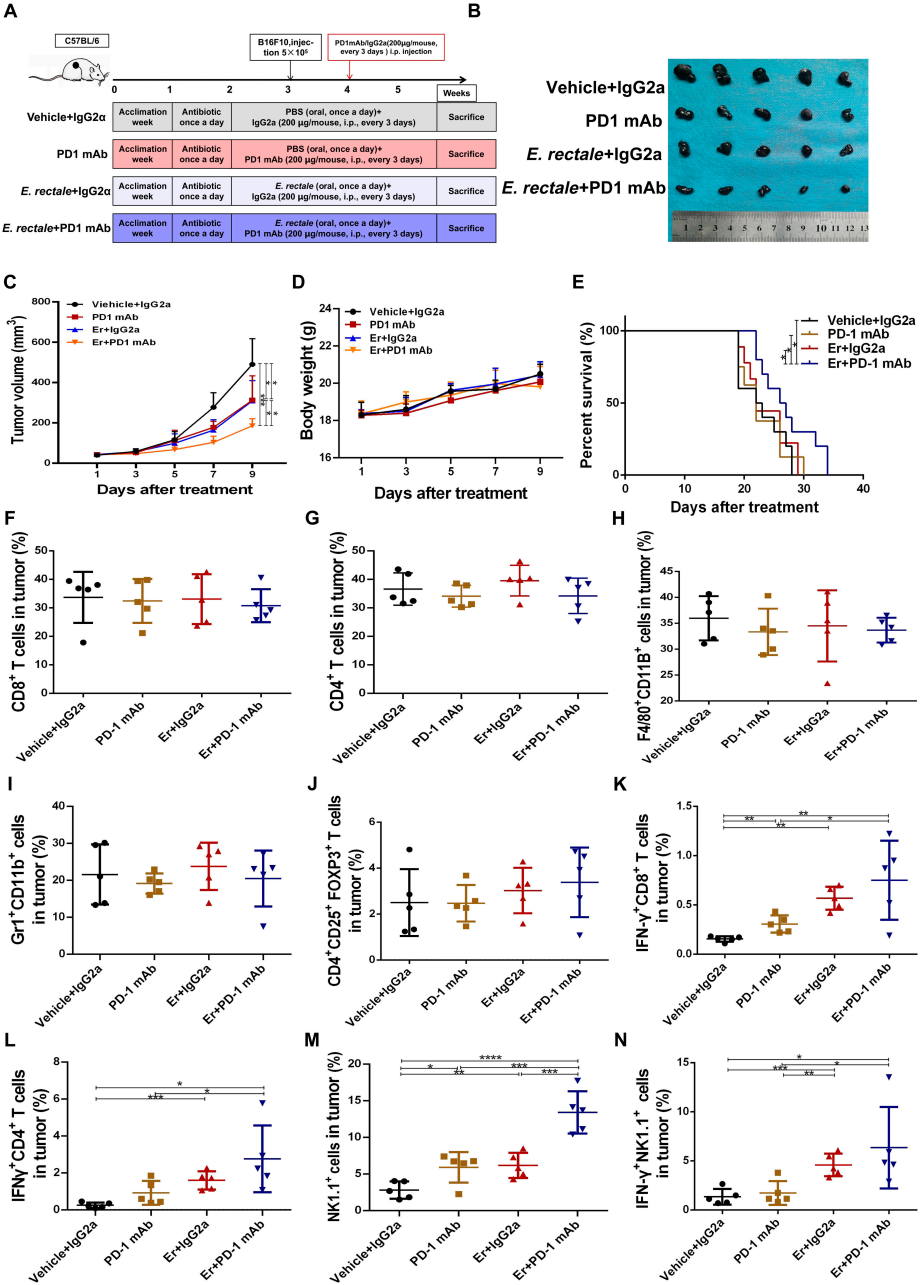
*E. rectale* enhances the efficacy of anti-PD1 treatment. (A) Experimental model of treatment of B16F10-bearing mice with *E. rectale* in combination with an anti-PD1 mAb. (B) Tumor samples isolated from C57BL/6 mice at the end of the treatment according to the administration method shown in the figure. (C) Tumor volume of mice measured every other day during the indicated treatment regimen. (D) Body weight of mice recorded every other day during the indicated treatment regimen. (E) Survival time (days) of mice beginning at tumor implantation (endpoints: the time at which the tumor volume reached 2,000 mm^3^ or death). (F to N) Flow cytometry detection of the CD3^+^CD8^+^ T cell proportion (F), CD3^+^CD4^+^ T cell proportion (G), F4/80^+^CD11B^+^ macrophage proportion (H), Gr1^+^CD11B^+^MDSC cell proportion (I), CD4^+^CD25^+^FOXP3^+^ T_reg_ cell proportion (J), IFN-γ^+^CD8^+^ T cell proportion (K), IFN-γ^+^CD4^+^ T cell proportion (L), NK1.1^+^ NK cell proportion (M), and IFN-γ^+^NK1.1^+^ NK cell proportion (N) in tumor tissue (*N* = 5). *E. rectale* is represented by *Er* in the figure*.* Multiple experimental data were counted and are presented according to the statistical method, and an asterisk (*) indicates the *P* value.

To study the role of *E. rectale* in anti-PD1 treatment, we constructed a tumor-bearing C57BL/6 mouse model colonized with *E. rectale* that was then treated with anti-PD1 immunotherapy (Fig. 2A). According to the reported doses administered per mouse, most bacteria were depleted before the oral gavage of bacterial species [23]. *ER* colonization was validated by polymerase chain reaction (PCR) after *E. rectale* administration or anti-PD1 treatment (Fig. S2A and B). As shown in Fig. 2B and C, application of *E. rectale* significantly enhanced the therapeutic efficacy of an anti-PD1 monoclonal antibody (mAb) (Fig. 2B and C) but did not affect mouse body weight (Fig. 2D). Additionally, consistent with the results for clinical patients shown in Fig. 1, *E. rectale* prolonged the survival of anti-PD1-treated mice (Fig. 2E). To explore the effect of the combination treatment on the immune microenvironment, we examined the proportions of various immune cells in tumors from treated mice by FCM. As shown in Fig. 2F to J, the infiltration of macrophages/MDSCs/T regulatory (T_reg_) cells in tumors was not dramatically different, indicating that these immune cells might not be involved in the enhancing effect of *ER* on the efficacy of PD1 mAb (Fig. 2F to J). However, interferon-γ (IFN-γ) expression in CD4^+^/CD8^+^ T cells was significantly increased in the combination treatment group, suggesting that the activity of T cells was enhanced (Fig. 2K and L). Importantly, the infiltration and activity of NK cells were both significantly increased in the combination treatment group (Fig. 2M and N), suggesting that *E. rectale* enhanced the therapeutic efficacy of anti-PD1 mAb by acting on NK cells.

### *E. rectale*-conditioned medium significantly increased NK cell activity

**Fig. 3.**
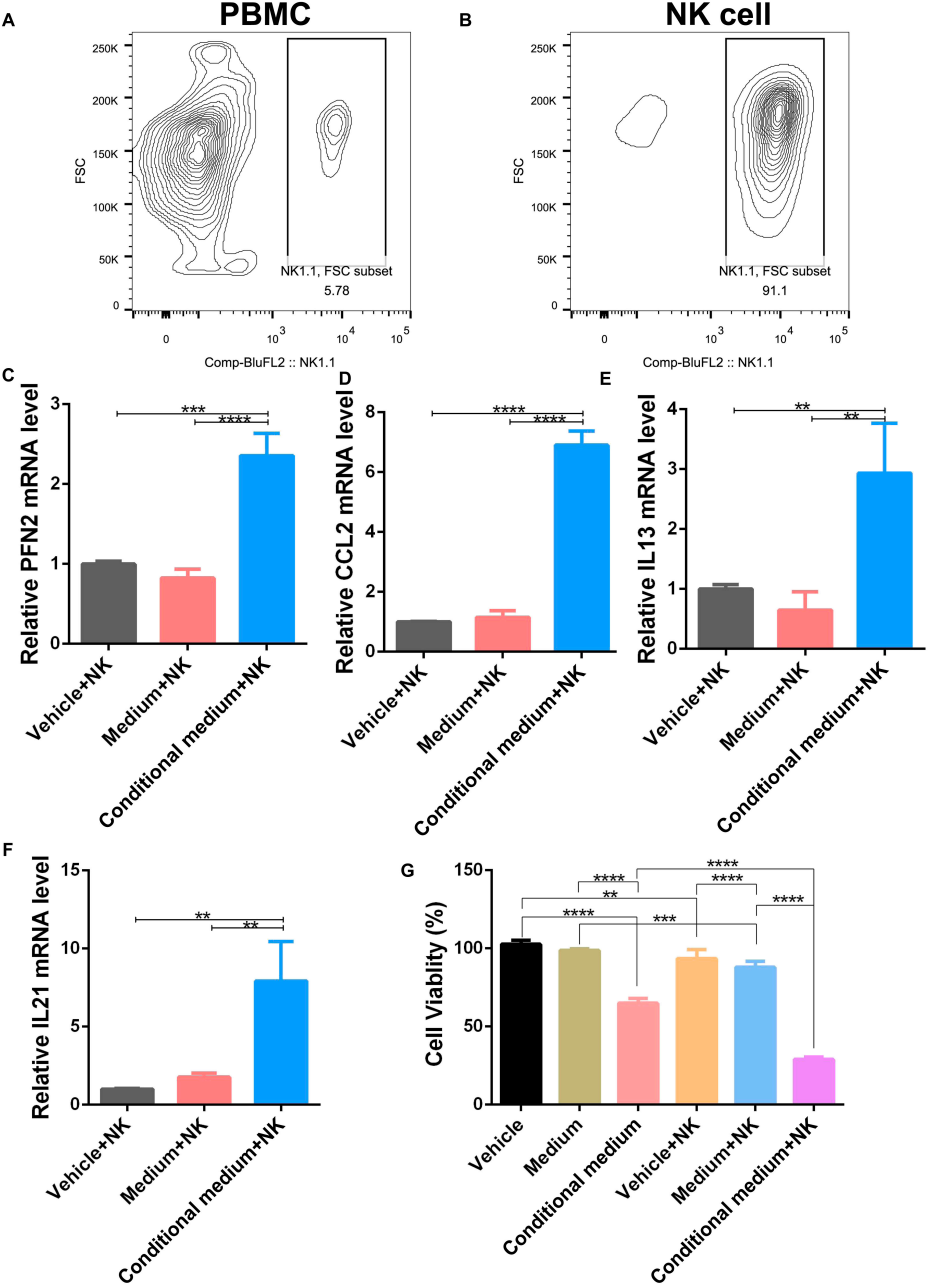
*E. rectale*-conditioned medium enhances the tumor-killing activity of NK cells. (A and B) Proportions of NK cells in mouse spleen peripheral blood mononuclear cells (PBMCs) before (A) and after (B) NK cell magnetic bead sorting. (C to F) RT-PCR analysis of differentiation and functional molecular indices (PFN2, CCL2, IL-13, and IL-21) of NK cells under vehicle, medium, and *E. rectale*-conditioned medium culture conditions. (G) Cytotoxic activity of NK cells against tumor cells under coculture conditions. Multiple experimental data were counted and are presented according to the statistical method, and an asterisk (*) indicates the *P* value.

It is well known that the gut microbiota affects host immunity by altering the metabolic microenvironment [24,25]. To study the role of *E. rectale* in NK cell function, we isolated primary NK cells from C57 mouse spleens using magnetic bead sorting (Fig. 3A and B) and treated the cells with *E. rectale*-conditioned medium, as shown in Fig. 3C to F. *E. rectale*-conditioned medium increased the expression of NK cell functional molecules, including PFN2, CCL2, IL-13, and IL-21 [5–7] (Fig. 3C to F). Furthermore, *E. rectale*-conditioned medium enhanced the NK cell killing of melanoma cells (Fig. 3G), suggesting that *E. rectale* might improve NK cell function by altering the microenvironment.

### Administration of *E. rectale* reduced l-serine production in vitro and in vivo

**Fig. 4.**
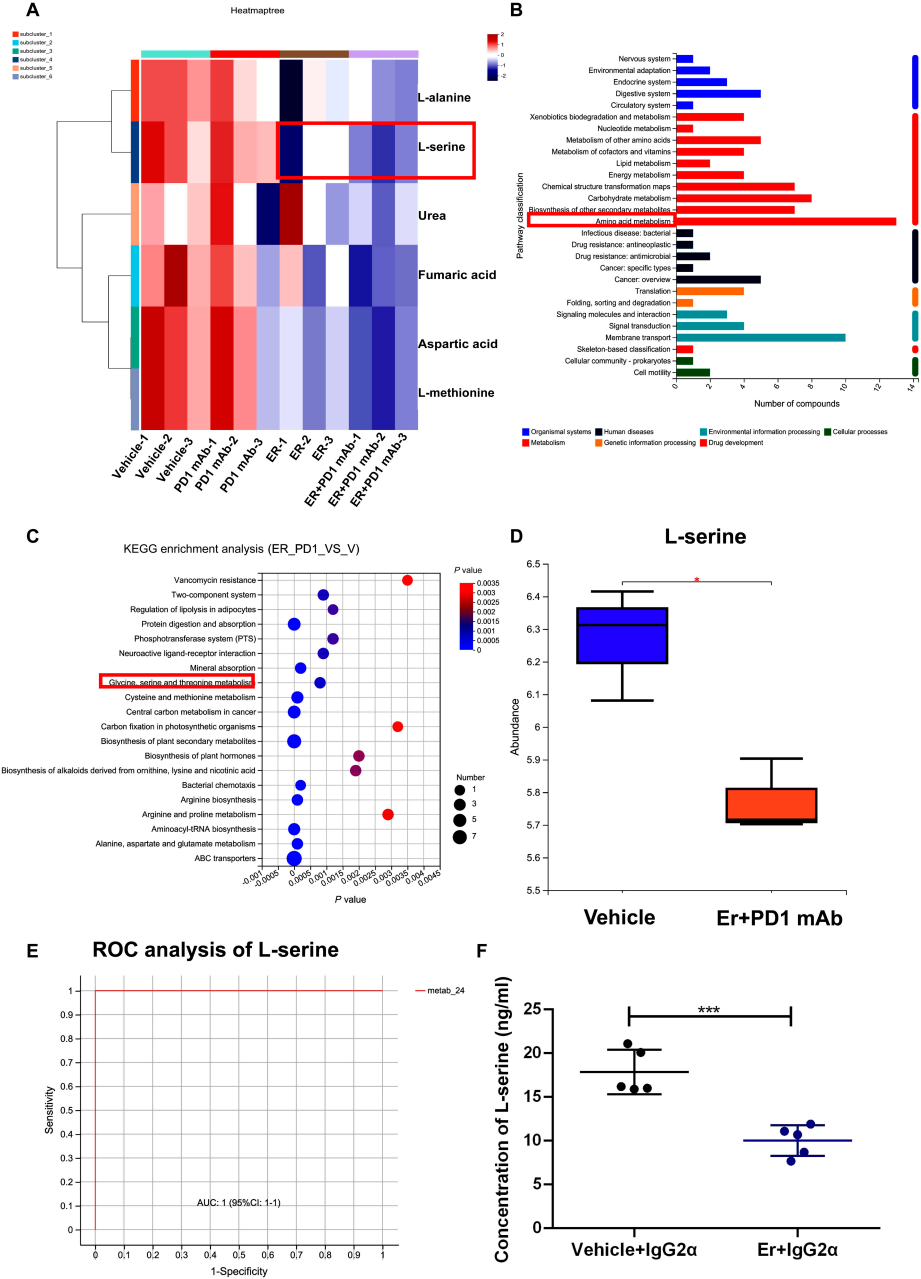
*E. rectale* reduced the l-serine in PD1 mAb-treated mice. (A) Heatmap of differential metabolites associated with l-serine detected by GC-MS metabolomics analysis. (B) KEGG pathway enrichment analysis of differential metabolites detected by GC-MS metabolomics analysis. (C) KEGG functional enrichment analysis of differential metabolites detected by GC-MS metabolomics analysis. (D) GC-MS metabolomics detected l-serine abundance in the vehicle and *E. rectale* plus anti-PD1 treatment groups. (E) Results for ROC analysis of l-serine in GC-MS metabolomics analysis. (F) UHPLC-MS/MS detected l-serine abundance in the serum of melanoma tumor-bearing mice treated with vehicle or *E. rectale*. *E. rectale* is represented by *Er* in the figure. Multiple experimental data were counted and are presented according to the statistical method, and an asterisk (*) indicates the *P* value.

To elucidate the mechanism by which *E. rectale* impacts the microenvironment of melanoma-bearing mice during anti-PD1 treatment, we collected gut feces from tumor-bearing mice at the end of anti-PD1 treatment and conducted metabolomic analysis to detect metabolite alterations in the feces. Principal component analysis (PCA) showed similar metabolic alterations in the control and anti-PD1 groups, while the *E. rectale* treatment alone and combination groups had similar patterns (Fig. S3A). Similarly, the classification effect determined by partial least squares discriminant analysis was more significant in the *E. rectale* and combination groups than in the other 2 groups (Fig. S3B). Furthermore, by analyzing the differentially expressed metabolites among the 4 groups, due to the dominant role of *E. rectale* in the feces, we found similar differentially expressed metabolites in the *E. rectale* and combination groups as well as in the control and anti-PD1 groups (Fig. S3C). Interestingly, administration of *E. rectale* markedly reduced the abundance of amino acid metabolites, especially l-serine, in the 2 groups with *E. rectale* application (Fig. 4A). Furthermore, by analyzing the classification of the Human Metabolome Database (HMDB) compounds of differential metabolites, we found that organic acids and derivatives accounted for the largest proportion (Fig. S3D). Similarly, KEGG functional enrichment analysis indicated that amino acid metabolism was dominant in the *E. rectale* application group (Fig. 4B). KEGG pathway enrichment analysis also indicated that the l-serine-related signaling pathway was the most significantly altered pathway (Fig. 4C); moreover, the abundance of l-serine was significantly decreased in the combination group compared with the control group (Fig. 4D). ROC analysis was utilized to calculate an AUC value of 1, indicating that the results of the metabolomic analysis were plausible (Fig. 4E). Then, we examined serum l-serine in *E. rectale*-colonized mice using the targeted metabolism method, and the results showed that the serum l-serine level in *E. rectale*-colonized mice was significantly lower than that in control mice (Fig. 4F). To investigate the relationship between *E. rectale* and l-serine metabolism, we performed genome-wide annotation of *E. rectale* [26–28]; the annotation showed that the genome of *E. rectale* encoded enzymes related to most amino acids and their derivatives (Fig. S3E), suggesting that consumption of amino acids is required for *E. rectale* survival. In addition, KEGG enrichment analysis of the *E. rectale* genome revealed that this genome encoded several enzymes related to the l-serine catabolic pathway (Fig. S3F), suggesting that *E. rectale* reduces the l-serine concentration in its environment by catabolizing l-serine. The depletion of l-serine by *E. rectale* was also found in the in vitro culture system (Fig. S3G). These results indicated that *ER* could reduce the abundance of l-serine by consuming environmental serine.

### Inhibition of l-serine synthesis enhanced anti-PD1 therapeutic efficacy through NK cell activation

**Fig. 5.**
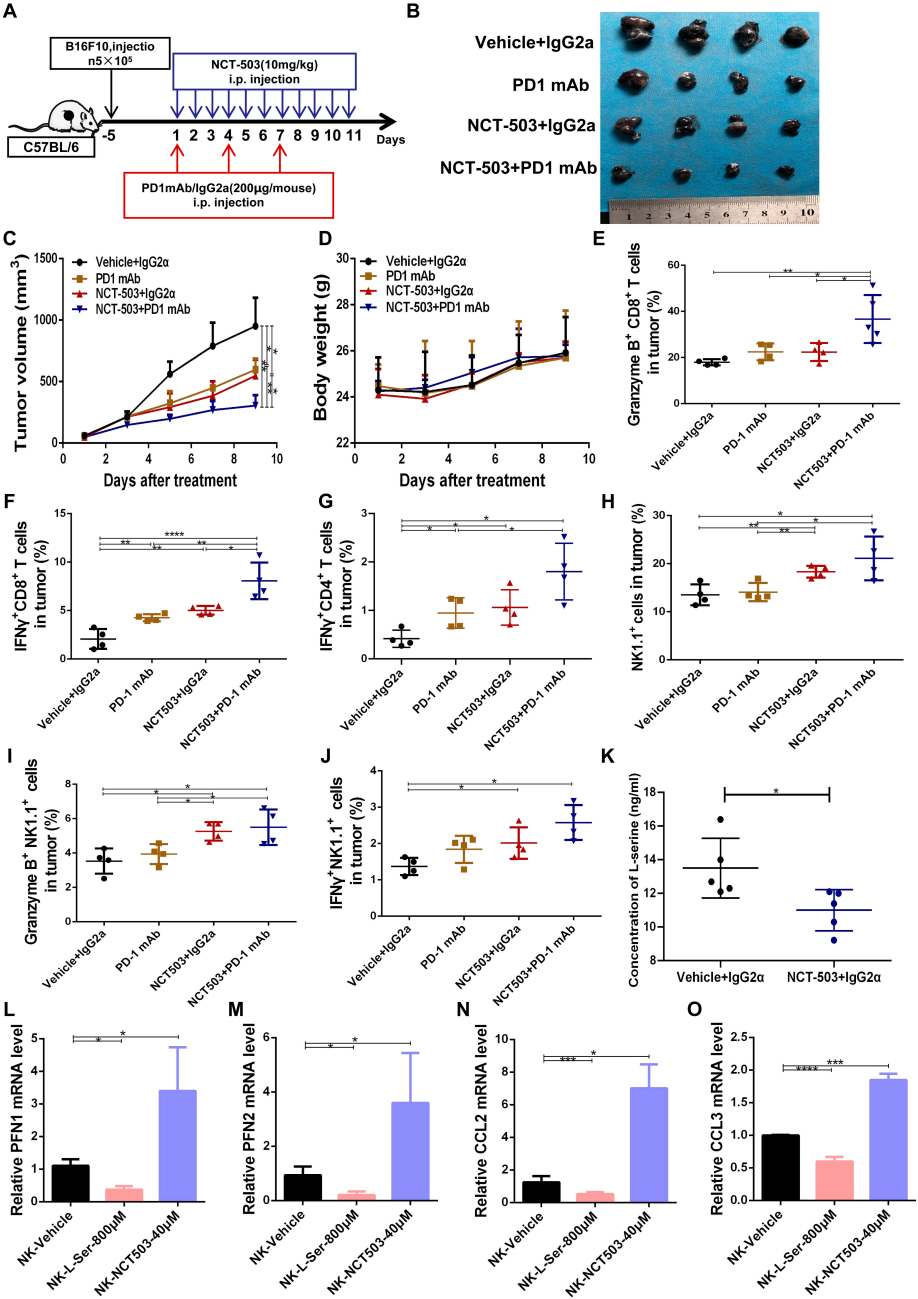
The l-serine synthesis inhibitor NCT503 enhances the efficacy of anti-PD1 treatment by affecting NK cells. (A) Model for the experimental method of using NCT503 combined with an anti-PD1 mAb to treat B16F10-bearing C57BL/6 mice. (B) Tumor tissues of mice collected at the end of the treatment regimen according to the administration method shown in the figure. (C) Tumor volume of mice measured every other day during the treatment regimen. (D) Body weight of mice recorded every other day during the treatment regimen. (E to J) Flow cytometry detection of the GZMB^+^CD4^+^ T cell proportion (E), IFN-γ^+^CD8^+^ T cell proportion (F), IFN-γ^+^CD4^+^ T cell proportion (G), NK1.1^+^ NK cell proportion (H), GZMB^+^NK1.1^+^ NK cell proportion (I), and IFN-γ^+^NK1.1^+^ NK cell proportion (J) in tumor tissues (*N* = 4). (K) Enzyme-linked immunosorbent assay (ELISA) detection of the serum l-serine concentration in vehicle- and NCT503-treated mice with melanoma. (L to O) RT-PCR detection of differentiation and functional molecular indices of NK cells under l-serine and NKT503 treatment. Multiple experimental data were counted and are presented according to the statistical method, and an asterisk (*) indicates the *P* value.

To verify whether *E. rectale* enhanced the efficacy of anti-PD1 immunotherapy by reducing l-serine availability, we used NCT503 to reduce the intracellular l-serine concentration, mimicking the depleting effect of *ER* on organismal l-serine. We treated B16F10 tumor-bearing mice with or without NCT503 [29], an inhibitor of the serine synthase PHGDH to inhibit l-serine synthesis, and with the anti-PD1 mAb (Fig. 5A). As shown in Fig. 5B to D, NCT503 significantly enhanced the therapeutic effect of the anti-PD1 mAb (Fig. 5B and C) and had no effect on body weight (Fig. 5D). Further analysis of the immune microenvironment revealed that NCT503 had effects similar to those of *E. rectale* administration and that NCT503 treatment favored the function of CD4^+^/CD8^+^ T cells and activity of NK cells (Fig. 5E to J). Targeted metabolic assays confirmed that NCT503 significantly reduced l-serine production in mice (Fig. 5K). To validate the effects of NCT503 and l-serine on NK cell function, we treated primary NK cells with l-serine or NCT503, and the findings showed that the application of l-serine inhibited the expression of functional and key molecules, including PFN1/2, CCL2, and CCL3, while NCT503 treatment enhanced NK cell activity (Fig. 5L to O). These results suggested that l-serine exerted a key role in anti-PD1 treatment in melanoma through effects on NK cell activity.

### l-Serine enhanced NK cell function through suppression of the FOS/FOSL2 signaling pathway

**Fig. 6.**
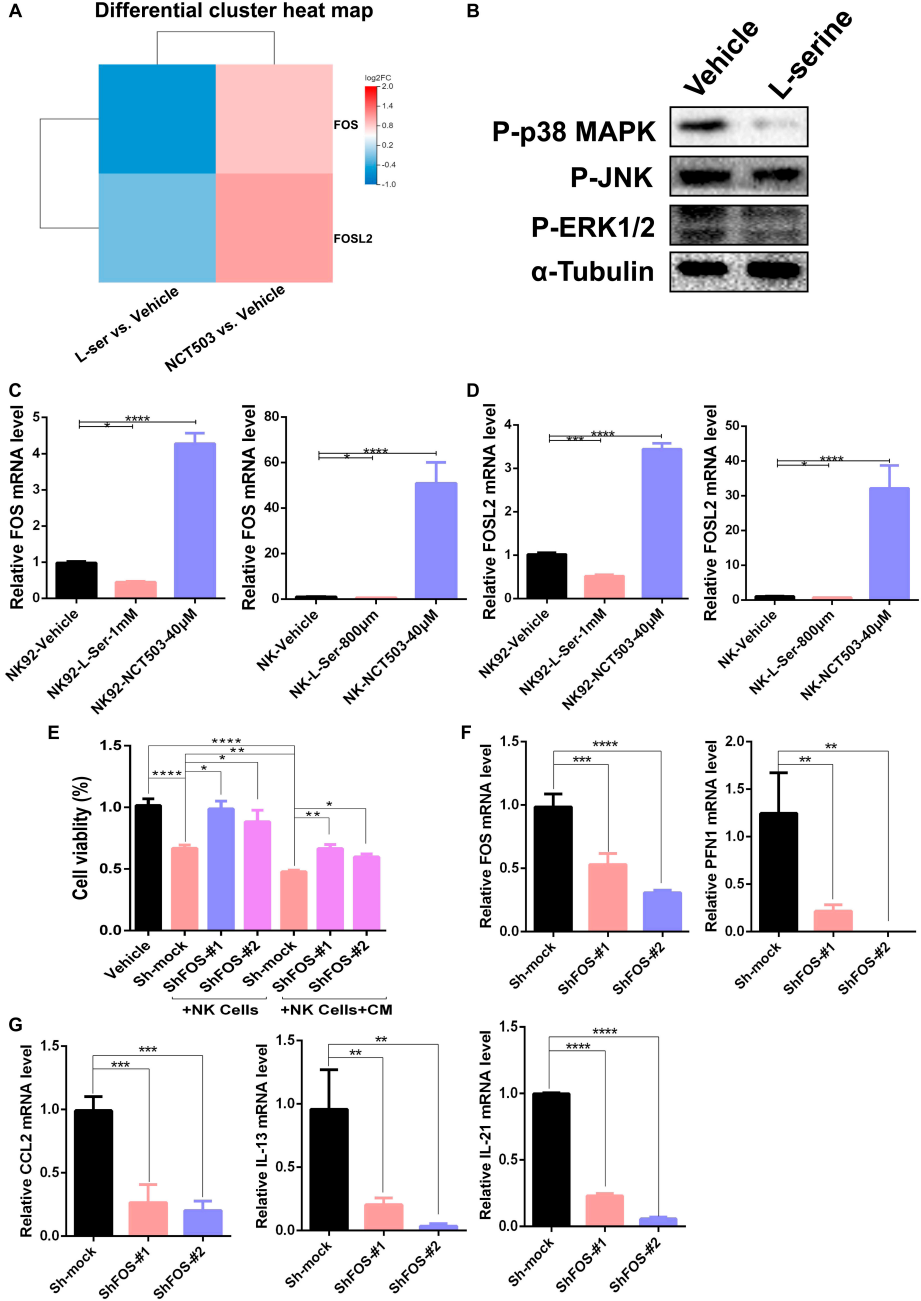
l-Serine inhibits NK cell function via the transcription factors FOS/FOSL2. (A) RNA-seq results shown in a differential gene expression heatmap for melanoma cells after serine or NCT503 treatment. (B) Western blotting detection of P-p38 MAPK expression in NK92 cells after l-serine (1 mM/l) treatment. (C and D) RT-PCR detection of FOS (C) and FOSL2 (D) mRNA expression in NK92 and primary NK cells under l-serine or NKT503 treatment. (E) Cytotoxic activity of NK92 cells against tumor cells after FOS knockdown and/or *E. rectale*-conditioned medium treatment. (F and G) RT-PCR detection of FOS, PFN1, CCL2, IL-13, and IL-21 mRNA expression after FOS knockdown in NK92 cells. Multiple experimental data were counted and are presented according to the statistical method, and an asterisk (*) indicates the *P* value.

To further elucidate the molecular mechanism by which l-serine impacts NK cell function, we performed RNA-sequencing (RNA-seq) analysis of NK cells to examine alterations in the transcriptional profile after l-serine or NCT503 treatment. We took the intersection of the 2 groups of differentially expressed genes and identified 73 similar differentially expressed genes (Fig. S4A). KEGG pathway enrichment analysis of the differentially expressed genes revealed that the mitogen-activated protein kinase (MAPK) pathway was significantly enriched (Fig. S4B). It was previously reported that activation of the MAPK pathway could increase the expression of NK cell-related functional molecules and thereby enhance NK cell killing [30,31]. Consistent with reported results, the expression of FOS and FOSL2, 2 key MAPK pathway-related transcription factors, was significantly up-regulated in the NCT503 treatment group but down-regulated after application of l-serine (Fig. 6A). Additionally, we confirmed at the protein level that l-serine has an inhibitory effect on the activation of MAPK signaling in NK cells (Fig. 6B). Moreover, quantitative reverse transcription PCR (qRT-PCR) verified the alterations in FOS and FOSL2 expression. The transcriptome sequencing results showed that the amount of l-serine was decreased in the NK92 cell line and primary NK cells after treatment with NCT503 or l-serine (Fig. 6C and D). Furthermore, knockdown of FOS impaired the cytotoxic effect of NK cells on melanoma cells and the promoting effect of *ER*-conditioned medium on NK cells (Fig. 6E). Moreover, knockdown of FOS expression significantly inhibited the expression of functional molecules in NK cells, including PFN1/2, CCL2, and CCL3 (Fig. 6F and G). Overall, these results revealed that l-serine inhibited NK cell function through FOS/FOSL2 signaling, whereas disruption of l-serine synthesis enhanced NK cell activity.

### Down-regulation of l-serine-related gene expression was associated with a reactive immune microenvironment and better responsiveness to anti-PD1 treatment

**Fig. 7.**
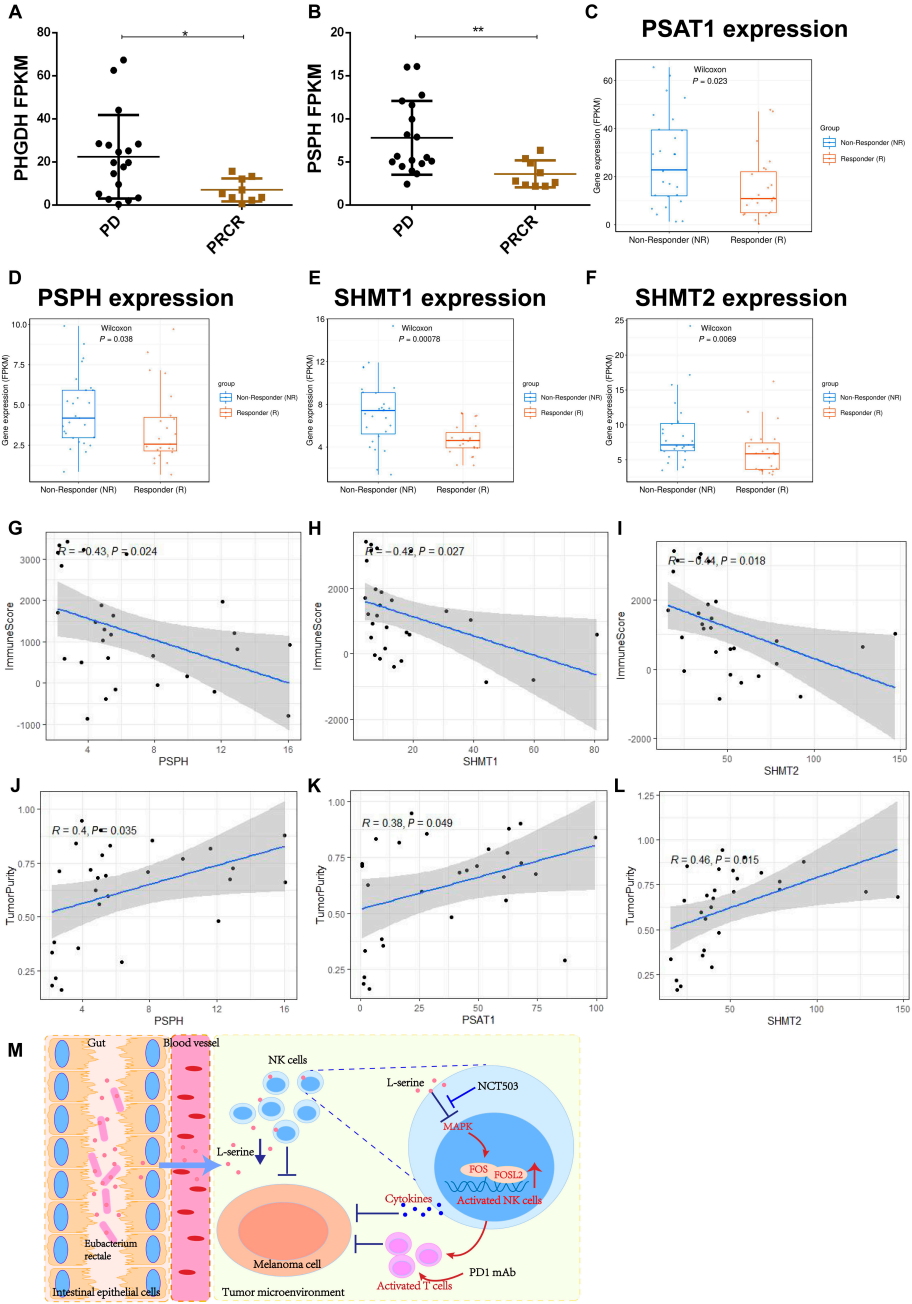
Correlation of l-serine synthesis pathway genes with anti-PD1 treatment efficacy. (A and B) Expression of the serine-metabolizing enzymes PHGDH (A) and PSPH (B) in anti-PD1-treated melanoma patients in the anti-PD1 treatment-nonresponsive (PD) group and anti-PD1 treatment-responsive (PRCR) group. (C to F) Expression of PAST1 (C), PSPH (D), SHMT1 (E), and SHMT2 (F) in anti-PD1-treated melanoma patients. (G to I) Correlations of PSPH (G), SHMT1 (H), and SHMT2 (I) with the immune score in PD1-treated patients. (J to L) Correlations of PSPH (J), PSAT1 (K), and SHMT2 (L) with tumor purity. (M) *E. rectale* promote NK cell function by reducing l-serine in the environment, which enhances the efficacy of PD1 immunotherapy. Multiple experimental data were counted and are presented according to the statistical method, and an asterisk (*) indicates the *P* value.

To verify the effect of l-serine on NK cell function, we constructed YUMM1.7 tumor-bearing mice responsive to anti-PD1 treatment (CR) and partially responsive B16F10 tumor-bearing mice (PR) and then performed single-cell sequencing of tumor tissues after anti-PD1 treatment. PHGDH, PSAT1, PSPH, and SHMT are key enzymes that regulate l-serine metabolism (Fig. S5A) [32]. As shown in Fig. S5B to E, the expression of PSAT1 and SHMT was significantly higher in the NK cell population of the PR group than in that of the CR group (Fig. S5B to E), while the expression of FOSL2 was significantly lower in the NK cells of the PR group than in those of the CR group (Fig. S5F and G). To verify the effect of l-serine on the efficacy of anti-PD1 immunotherapy, we also downloaded RNA-seq data (GSE 91061) for clinical anti-PD1-treated melanoma patients from the Gene Expression Omnibus (GEO) database, which showed that the expression of PHGDH and PSPH was significantly lower in patients who achieved a PR or CR in response to anti-PD1 treatment than in drug-resistant patients with PD (Fig. 7A and B). Similarly, in the PRJEB23709 melanoma anti-PD1 immunotherapy cohort [33], the levels of PSAT1, PSPH, and SHMT1/2 were significantly reduced in PR or CR patients treated with anti-PD1 compared with drug-resistant PD patients (Fig. 7C to F). We further analyzed immune cell enrichment in the immune microenvironment of anti-PD1-treated patients using the ImmuneCellAI-human method [34] and the association between T cell enrichment in the tumor microenvironment and l-serine metabolism. As shown in Fig. S6A to C, the abundance of l-serine metabolic enzymes (PSAT1, PSPH, and SHMT1) was negatively correlated with CD8^+^ T cell infiltration (Fig. S6A to C). PSAT1 and SHMT1/2 were negatively correlated with CD4^+^ T cell infiltration (Fig. S6D to F). PSPH and SHMT1 were also negatively correlated with gamma_delta T cell infiltration (Fig. S6G to I). These results indicated that the l-serine metabolic signaling pathway was associated with a suppressive tumor immune microenvironment. To further validate our conclusions, we used the ESTIMATE algorithm to assess the immune microenvironment of anti-PD1-treated patients and found that the expression of PSPH and SHMT1/2 was negatively correlated with patient immune scores (Fig. 7G to I), while PSAT1, PSPH, and SHMT2 were positively correlated with tumor purity (Fig. 7J to L). Additionally, lower expression of SHMT1/2 was related to longer survival in melanoma patients treated with anti-PD1 immunotherapy (Fig. S6J and K).

In conclusion, intestinal *E. rectale* enhanced NK cell function and anti-PD1 therapeutic efficacy by consuming l-serine in its environment, leading to the activation of NK cell activity through the FOS/FOSL2 signaling pathway (Fig. 7M). Therefore, *E. rectale* transplantation could be a novel approach to improve the efficacy of anti-PD1 immunotherapy in melanoma.

## Discussion

Mechanisms of primary and acquired resistance to ICIs have been characterized by genetic susceptibility, including a lack of antigen processing or abnormal antigen processing, T cell rejection or T cell depletion, PD-L1 expression, extrinsic factors such as microorganisms, and host factors [35–37]. Among these factors, gut microbes have been shown to affect the efficacy of ICI therapy by altering the proportions and activities of immune cells, such as CD4^+^ T cells, CD8^+^ T cells, T_reg_ cells, NK cells, and macrophages. The detailed effects of gut microbes include the following: (a) in regard to enhancement of the host effector immune response, supplementation with Bifidobacterium enhanced the efficacy of an anti-PD-L1 mAb by activating DCs and inducing CD8^+^ T cell killing [38]. (b) In regard to enzymes or surface antigen structures, the enzyme SagA expressed by *Enterococcus faecalis* activated innate immunity by releasing peptidoglycan fragments, inducing a microenvironment favorable for immunotherapy and that enhanced the immunotherapeutic effects [13]. The presence of a major histocompatibility complex I (MHC-I) binding epitope [TSLARFANI (TMP1)] in *Enterococcus haii* that can cross-react with TMP in tumors resulted in improved immunotherapeutic effects by the generation of TMP-specific H-2Kb-restricted CD8^+^ T-lymphocyte responses that enhanced the efficacy of an anti-PD1 mAb [39]. (3) For metabolite regulation, *Bifidobacterium pseudolongum*-derived inosine increased the efficacy of ICIs in colon cancer by acting on inosine 2A receptors on T cells to enhance the antitumor immune response [40]. Pectin, a soluble dietary fiber, increased butyric acid production in the feces of mice by altering the intestinal microbial community and thereby enhanced the efficacy of an anti-PD1 mAb in colorectal cancer [41].

In melanoma, interventions targeting the intestinal flora are an effective strategy to improve the efficacy of immunotherapy; therefore, the identification of probiotics and clarification of mechanisms will improve the understanding of the role of the microbiota in immunotherapy in melanoma. In this study, we found that *E. rectale* was significantly enriched in the intestine of patients who responded to ICI therapy, especially those who responded to anti-PD1 immunotherapy (Fig. 1 and Fig. S1). Melanoma patients with a high abundance of *E. rectale* had prolonged overall survival (Fig. 1 and Fig. S1). We further confirmed that *E. rectale* promoted anti-PD1 immunotherapy efficacy in a preclinical model (Fig. 2).

Mechanistically, administration of *E. rectale* significantly increased the infiltration and killing activity of NK cells in vitro and in vivo (Figs. 2 and 3). NK cells not only are important innate immune cells but also regulate the functions of various immune cells through the release of Fms-related tyrosine kinase 3 ligands and the chemokines CCL5 and XCL1, ultimately initiating the activation of CD8^+^ T cells [42]. Evidence shows that ICI treatment reduces tumor volume even in the absence of T cells in mice, implying that other immune cells, such as NK cells, play important roles in ICI treatment [43]. Interestingly, pluripotent stem cell-derived NK cells in combination with an anti-PD1 mAb were found to produce relatively high levels of inflammatory cytokines, which enhance the antitumor effects [42]. A clinical study showed that the objective remission rate achieved with combination treatment of NK cells and an anti-PD1 mAb reached 36.5%, compared to a rate of 18.5% for the anti-PD1 mAb alone, and this combination treatment significantly prolonged overall survival in non-small cell lung cancer [44]. In melanoma, metformin activates NK cells in a MAPK-dependent manner and further increases the anti-PD1 immunotherapeutic efficacy [30]. Consistent with above results, FOSL2 [a component of the MAPK downstream transcription factor activator protein 1 (AP1)] promotes NK maturation and function [45]. The natural cytotoxicity exerted by human NK cells is associated with AP1 transcription factor genes such as JunB, FosB, and c-Fos [46]. In melanoma, microRNAs in NK cells promote the clearance of B16F10 melanoma in vivo by NK cells through activation of nuclear factor κB (NF-κB) and AP1 [47]. However, the mechanism of regulation of NK cells by *E. rectale* in melanoma is not clear.

To further elucidate the molecular mechanisms by which *E. rectale* acts on NK cells, we analyzed the whole genome of *E. rectale*, which was found to contain several genes encoding serine-catabolizing enzymes that could consume serine from the environment. Consistent with this analysis, our metabolic results showed that administration of *E. rectale* dramatically reduced the abundance of l-serine. l-Serine, a nonessential amino acid, is involved in protein, nucleotide, and lipid synthesis; provides one-carbon units for the folate cycle and methylation reactions; and is a nutrient required for tumor growth [48]. An elevated l-serine level has been associated with tumor disease progression, a poor prognosis for tumor patients and resistance to BRAF inhibitors in melanoma, pancreatic cancer, and non-small cell lung cancer cells [49]; meanwhile, inhibition of l-serine synthesis significantly reduce tumor cell numbers [50]. Additionally, serine metabolism has been shown to affect immune cell activity. For macrophages, high levels of serine metabolites suppress M1 macrophage production of IFN-I via YAP-mediated blockade of the TBK1–IRF3 axis, while restriction of serine production enhances IFN-β-mediated innate immunity in vitro and in vivo [51,52]. In mouse embryonic fibroblasts, inhibition of serine synthesis leads to MAPK pathway activation [53]. In addition, serine helps maintain the mitochondrial fusion–fission balance and inactivates MAPK to protect neuronal cells from oxidative stress [54].

Here, we found that the serine synthesis inhibitor NCT503 significantly enhanced anti-PD1 immunotherapeutic efficacy and NK cell killing activity in vitro and in vivo. Moreover, high expression of enzymes related to serine metabolism correlated with nonresponsiveness to immunotherapy in melanoma patients; therefore, we concluded that administration of *E. rectale* led to the consumption of serine, which activated NK cell activity and consequently improved the efficacy of anti-PD1 immunotherapy in melanoma. In addition, PHGDH, PSAT1, PSPH, and SHMT are key enzymes related to serine metabolism [55]. Among these enzymes, PHGDH is the first rate-limiting enzyme in serine synthesis and the gatekeeper of serine synthesis [56]. PHGDH is highly expressed in a variety of tumors and positively related to drug resistance. NCT503 was identified from the National Institutes of Health (NIH) Molecular Libraries Small Molecule Repository (MLSMR) drug library as a specific inhibitor of PHGDH [57]; it inhibited the malignant phenotype in breast, liver, and lung cancers in a PHGDH-dependent manner [55–57]. In this study, our findings confirmed that NCT503 significantly reduced serine production and increased NK cell killing activity. Moreover, we confirmed the sensitizing effect of NCT503 on melanoma treated with anti-PD1 immunotherapy, suggesting that both NCT503 and *E. rectale* could be used as novel agents to improve the efficacy of anti-PD1 immunotherapy in melanoma. Overall, we elucidated the role of the *E. rectale–*serine–NK cell axis in anti-PD1 immunotherapy, which provides a promising therapeutic strategy for improving the efficacy of anti-PD1 immunotherapy in melanoma.

## Materials and Methods

### Gut flora cohort analysis

A cohort of fecal macrogenomic data on the efficacy of melanoma treatment with ICIs was downloaded from the National Center for Biotechnology Information (NCBI) database. Clinical information of the included samples is shown in Table S1 (cohort 1) and Table S2 (cohort 2). Data quality control, host data removal, and species diversity analysis were re-performed, followed by LEfSe analysis and survival analysis. Further, to validate the results of the analysis, logistic regression models were first constructed in the training set based on the relative abundance of microbial species versus patient phenotype data, and then the predictive performance was estimated in the testing set using ROC curves.

### Experimental cells and bacterial strains

Murine-derived melanoma cell line B16F10 was obtained from the American Type Culture Collection; B16F10 cells were cultured in RPMI 1640 (Gibco, USA) medium containing 10% fetal bovine serum (Gibco, USA); NK92 cells were obtained from Meisen Chinese Tissue Culture Collections (Meisen CTCC, China) and cultured in Alpha Minimum Essential medium containing 12.5% horse serum and 200 U/ml recombinant IL-2 (Meisen CTCC). All cells were cultured in an anaerobic incubator with 5% CO_2_ at 37 °C. *E. rectale* DSM 17629 bacterial strains were obtained from the Deutsche Sammlung von Mikroorganismen und Zellkulturen (DSMZ); the strains were cultured in an anaerobic incubator using PYG (PYG Broth Medium Base) medium (Mingzhoubio, China).

### Animal models

Six- to 8-week-old female C57BL/6 mice were purchased from SLAC Laboratory Animal Co. Ltd. The animals were housed in the specific pathogen-free environment of the Department of Laboratory Animal Science, Central South University. The animal experiments were approved by the Animal Ethics Committee of Central South University and conducted in compliance with the “3R” principle.

To construct *E. rectale* colonized mice, first, 4 antibiotics [25 mg/kg vancomycin (Sangon Biotech, China), 50 mg/kg metronidazole (Sangon Biotech), 50 mg/kg ampicillin (Sangon Biotech), and 50 mg/kg neomycin (Sangon Biotech)] were used for gavage and purging for 1 week; then, 200 μl (1.0 × 10^10^ colony-forming units/ml) of *E. rectale* solution resuspended in phosphate-buffered saline (PBS) was used for gavage for 1 week. The fecal samples were collected before and after gavage, DNA was extracted using the QIAamp DNA Stool Mini Kit (QIAGEN, Germany), and then qRT-PCR was performed to detect whether *E. rectale* were successfully colonized. After the successful colonization, 100 μl of B16F10 cells containing 5 × 10^5^ cells was implanted under the skin of the right dorsal side of C57BL/6 mice. When the tumor size reached about 20 to 30 mm^3^, 200 μg of PD1 mAb (BioXCell, BE0146, USA) was given in the anti-PD1 treatment group and *E. rectale* + anti-PD1 groups every 3 days. Mice in the *E. rectale* group were kept in *E. rectale* gavage daily during the experiment. For l-serine inhibitor experiments, vehicle [corn oil (Aladdin, China)] + 200 μg of IgG2a (BioXCell, BE0089, USA), 200 μg of PD1 mAb (BioXCell, BE0146, USA), 10 mg/kg NCT503 + 200 μg of IgG2a, and 200 μg of PD1 mAb + 10 mg/kg NCT503 (Selleck, USA) were given in groups when the tumor grew to 20 to 30 mm^3^; tumor volume was measured by vernier calipers every other day, and body weight was measured every other day. When the tumor grew to nearly 1,000 mm^3^, the tumor, serum, and feces were taken from mice for follow-up experiments.

### Multicolor flow cytometry

The cells or tumor tissues were prepared as single-cell suspensions; 100 μl of prepared Zombie Aqua Fixable Viability Kit (anti-BV510, BioLegend, USA) was added and incubated for 10 to 15 min at room temperature; configured CD16/32 (BioLegend) was added, incubated for 15 min at 4 °C, and protected from light; and 100 μl of prepared surface antibody [APCCY7-CD45 antibody, APC-CD3 antibody, PE-Cy5.5-CD4 antibody, PE-Cy7-CD8 antibody, PE-NK1.1, PE-Cy5.5-CD25, PE-Cy5.5-Gr-1, APC-F4/80 antibody, and PE-CD11B antibody (BioLegend)] was added and incubated for 30 min at 4 °C. To label intracellular molecules, cells were incubated for 30 min from light after fixation and permeabilization (eBioscience, USA); 100 μl of the prepared intracellular antibody [FITC-Granzyme B antibody (BioLegend), PE-FOXP3 antibody (eBioscience), and BV711-IFN-γ antibody (BioLegend)] was added and incubated for 30 min at 4 °C. The stained cells were analyzed by FACS LSRFORTESSA (BD, USA), and the data were analyzed by FLOWJO software.

### NK cell isolation

The lymphocytes in the spleen of C57BL/6 mice were isolated by gradient separation of mouse lymphocyte isolation solution (DAKEWE, China), and then the primary NK cells were sorted by NK cell isolation kit (Miltenyi, Germany) according to the instructions.

### Coculture assay

Tumor cells and NK cells were grown in a 96-well plate at a ratio of 1:2.5, *E. rectale*-conditioned culture supernatant (1:40) was added, and a control group without NK cells or *E. rectale*-conditioned culture supernatant was set up. The 96-well plates were incubated in an incubator at 37 °C with 5% CO_2_ for 48 h and then tested for cell survival by CCK8 kit (Selleck, USA). The cell survival was then calculated using the control group as a reference.

### Cell proliferation assay

The tumor cells or coculture cells were grown in 96-well plates and washed twice with PBS after 48 h; 100 μl of fresh complete medium containing CCK8 kit (Selleck, USA) in 10:1 configuration was added and incubated in a cell incubator containing 5% CO_2_ at 37 °C for 1.5 h; and then the absorbance value of each well at 450 nm was measured by Microplate Reader (BioTek, USA).

### Quantitative reverse transcription PCR

RNA was extracted by lysis with Tripure (Bio Teke, China), trichloromethane (SCR, China), isopropanol (SCR), and 75% alcohol (SCR); cDNA was synthesized by reverse transcription kit (Yeasen, China); cDNA was amplified and analyzed by Ultra SYBR Mixture (Bimake, China) on a qRT-PCR-Q3 system (Applied Biosystems, USA). PCR primers are listed in Table S3.

### Gas chromatography–mass spectrometry and ultrahigh performance liquid chromatography–tandem mass spectrometry

Gas chromatography–mass spectrometry (GC-MS) is done by Shanghai Majorbio Technology Co. In brief, a 50-mg frozen stool sample is ground, metabolites are extracted and derivatized, GC-MS analysis information is extracted, and data are normalized, standardized, and then subjected to multivariate statistical analysis, differential metabolite analysis, and functional analysis.

Ultrahigh-performance liquid chromatography (AB SCIEX UHPLC, USA) coupled with a triple-quadrupole mass spectrometer (Triple Quad 5600, USA) was applied for l-serine analysis. Twenty microliters of serum sample was diluted 10 times with saline; 60 μl of methanol containing serine internal standard was added, vortexed for 5 min, and centrifuged at 4 °C, 12,000 rpm for 10 min; 10 μl of supernatant was put into a new EP tube; and sodium borate solution and 6-aminoquinolinyl-*N*-hydroxysuccinyl carbamate were added. Mixed well and incubated in a water bath at 55 °C for 10 min. The supernatant was aspirated, diluted with pure water, and vortexed for 5 min. The diluted samples were centrifuged at 4 °C for 5 min at 12,000 rpm; 50 μl of supernatant was taken for analysis [58].

### Transcriptomics

The RNA-seq assay is done by Wuhan Huada Sequencing Company. First, mRNA enrichment and purification: oligo dT selection to enrich the mRNA; RNA fragmentation and cDNA synthesis (second-strand cDNA synthesis with dUTP instead of dTTP); end repair, add A and adaptor ligation; PCR; circularization and make DNB; sequencing on DNBSEQ platform. Subsequently, the data obtained from sequencing are subjected to quality control (QC) [59], and clean reads are obtained to compare to the reference sequence [60]. If the second QC (QC of alignment) is passed, subsequent differential gene analysis and functional analysis will be performed.

### Statistics

Experimental data analysis was performed using GraphPad software (version 6.01), and experimental data were expressed as mean ± SD. The chi-square of the data was tested, *t* test was used for comparison between 2 groups with chi-square, one-way analysis of variance (ANOVA) was used for comparison between multiple groups with chi-square, and nonparametric test was used for non-chi-square. For the survival data analysis, the Kaplan–Meier method and Gehan–Breslow–Wilcoxon test were used to detect difference in survival curves between groups. Statistical significance was achieved when *P* < 0.05, and the asterisk represents the degree of difference (*P < 0.05; **P < 0.01; ***P < 0.001; ****P < 0.0001).

## Data Availability

The accession number for the raw RNA-seq data reported in this paper is GEO: GSE225920. The datasets analyzed during the current study are available from the corresponding author on reasonable request.
