# Supplementary material for: Eubacterium rectale Improves the Efficacy of Anti-PD1 Immunotherapy in Melanoma via l-Serine-Mediated NK Cell Activation
Source: Research (Wash D C). 2023 Apr 28;6:0127. doi: 10.34133/research.0127 (PMC10202379; doi:10.34133/research.0127)
Supplement: Supplementary 1 — Figs. S1 to S6 Tables S1 to S3 [file research.0127.f1.doc]

Supplementary Materials:

**Materials and Methods**

**Single cell sequencing and analysis**

The tumors from B16F10_anti-PD1 and Yumm1.7_anti-PD1 were prepared as single cell suspensions for counting, viability and concentration detection by Scanner. The single cell suspensions were injected into Cartridge for cell sedimentation. Subsequent capture of mRNA and antibody tags is performed using Poly-dT on magnetic beads. A cDNA library is constructed from the mRNA on the magnetic beads. At the same time, library construction is performed on Sample Tag on magnetic beads. Data comparison and data statistics were performed by matching and splitting the CL and UMI sequences on the two library sequences. Subsequently, BD genomics rhapsody was used for scRNA-Seq gene expression quantification based on UMI counts. Seurat software was used for data quality control and filtering: the number of genes identified in the cells was filtered out from less than 200 or greater than 90% of the maximum number of genes; the top 15% of mitochondrial reads were filtered out; and the effect of cell cycle was corrected; Seurat and Doubletdetection software were used for high variable features screening and subsequent dimensionality reduction analysis, multiplex data integration, difference analysis, pathway enrichment, etc.

**Supplementary Figures:**


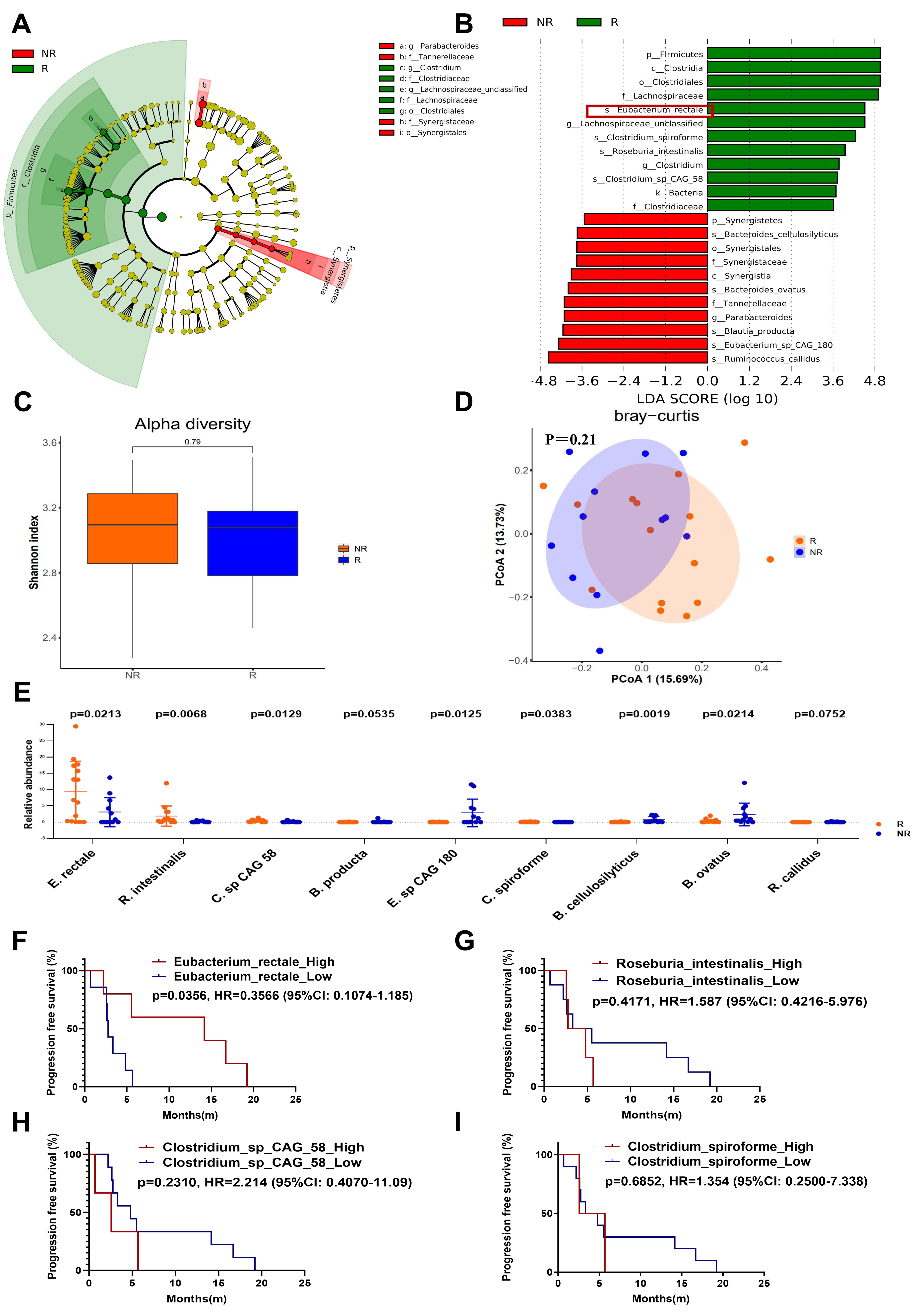


Figure S1: Analysis of gut microbial composition in patients treated with ICI for melanoma. A-B. Results of LEfSe analysis for the R and NR groups. (A) Evolutionary branching plots indicate differences in abundance for different species. (B) Bar graphs represent the LDA values of specific species with a significant difference in abundance between the R and NR groups (LDA ≥ 3, P < 0.05). C. Results of alpha diversity based on the Shannon index; D. PCoA (Principal co-ordinates analysis) plot showed the results of beta diversity analysis based on the Bray Curtis algorithm. E. Comparison of relative abundance of nine species in the R and NR groups (Wilcoxon test). F-I. Survival curves of patients corresponding to *Eubacterium rectale* (F), Roscoe intestinalis (G), Clostridium sp CAG 58 (H), and Clostridium spiroforme (I) abundance that were significantly enriched in the R group. R, treatment-responsive; NR, (treatment-nonresponsive); R (n=15), NR (n=12). Multiple experimental data were counted and are presented according to the statistical methods, and an asterisk (*) indicates the p value.


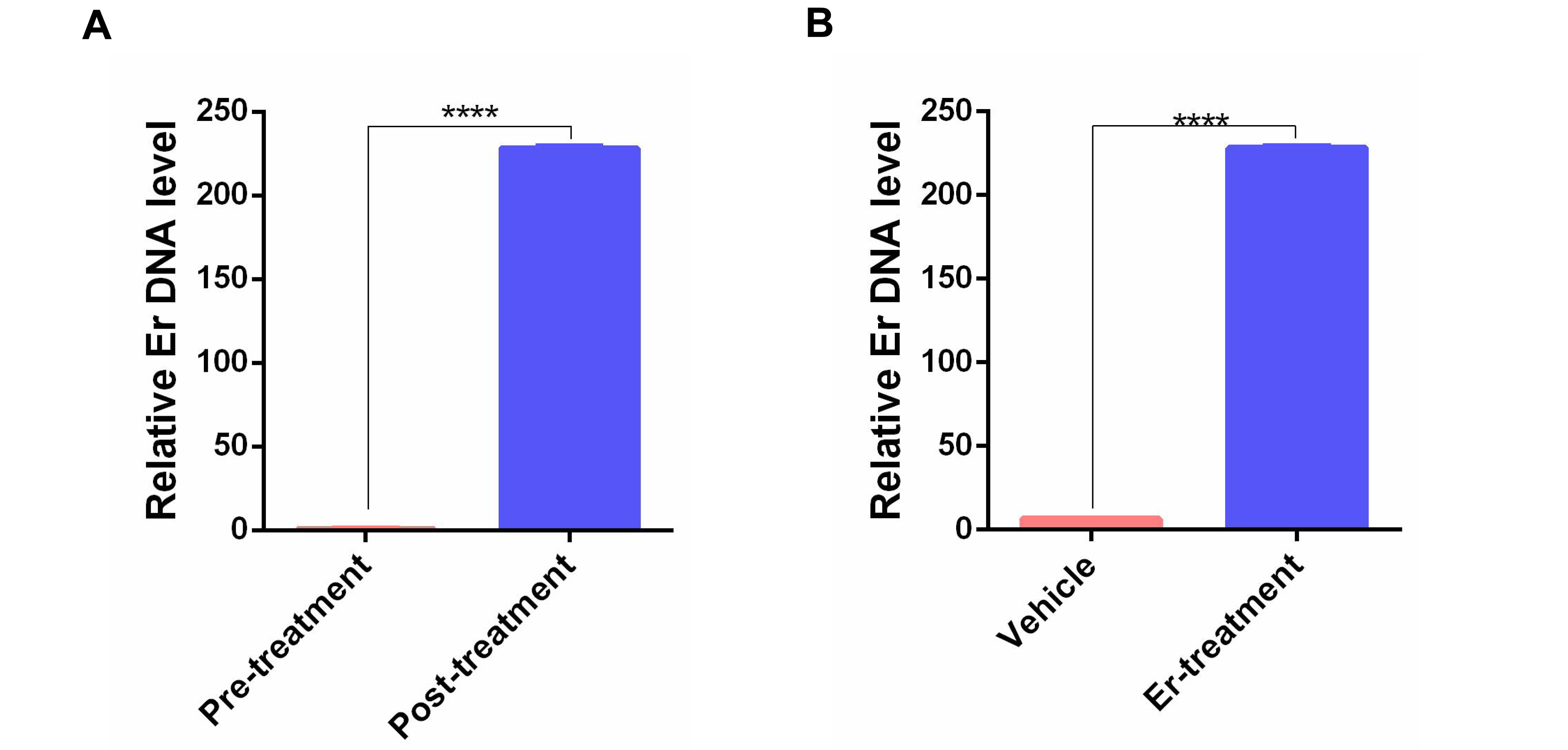


Figure S2: Detection of intestinal colonization of *E. rectale*. A. DNA abundance of *E. rectale* in mouse intestinal feces detected by qRT-PCR before and after *E. rectale* colonization. B. DNA abundance of *E. rectale* in mouse intestinal feces detected by qRT-PCR before and after anti-PD1 treatment. *E. rectale* is represented by *Er* in the figure. Multiple experimental data were counted and are presented according to the statistical methods, and an asterisk (*) indicates the p value.


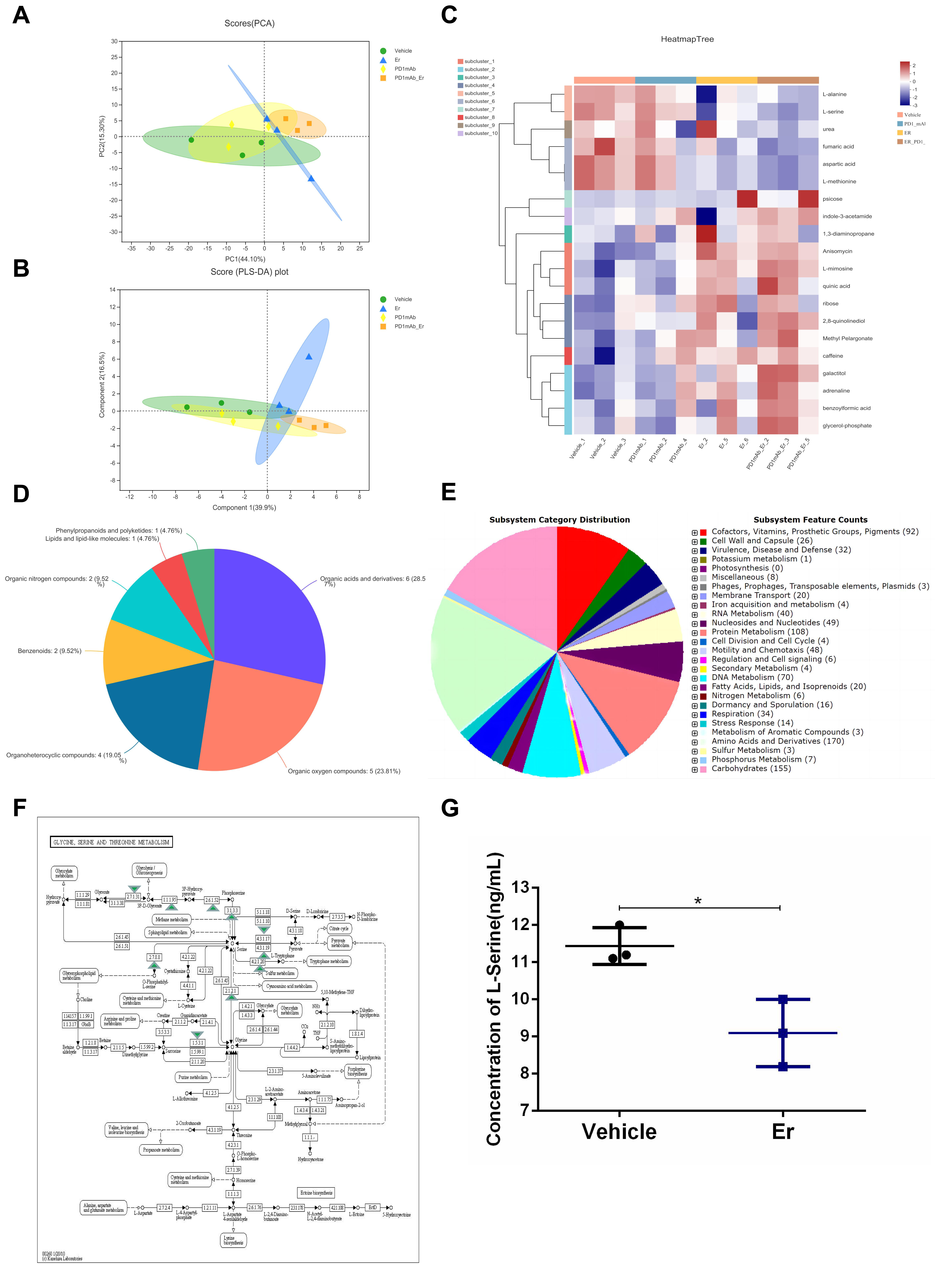


Figure S3: *E. rectale* reduce L-serine in the environment. A. Plot of PCA analysis results; B. Plot of PLS-DA analysis results. C. Heatmap of the top 20 differentially metabolized substances detected by metabolomic analysis. D. Classification of differentially metabolized substances in the Human Metabolome Database (HMDB). E. Functional distribution of genes encoded by *E. rectale* genome; F. KEGG metabolic pathway of genes encoded by *E. rectale* genome Annotation diagram: boxes are labeled with enzymes involved in L-Serine metabolism, and green triangles indicate *E. rectale* encoded enzymes. G. ELISA detection of L-serine concentrations in the medium with or without *E. rectale*. *E. rectale* is represented by *Er* in the figure. Multiple experimental data were counted and are presented according to the statistical method, and an asterisk (*) indicates the p value.


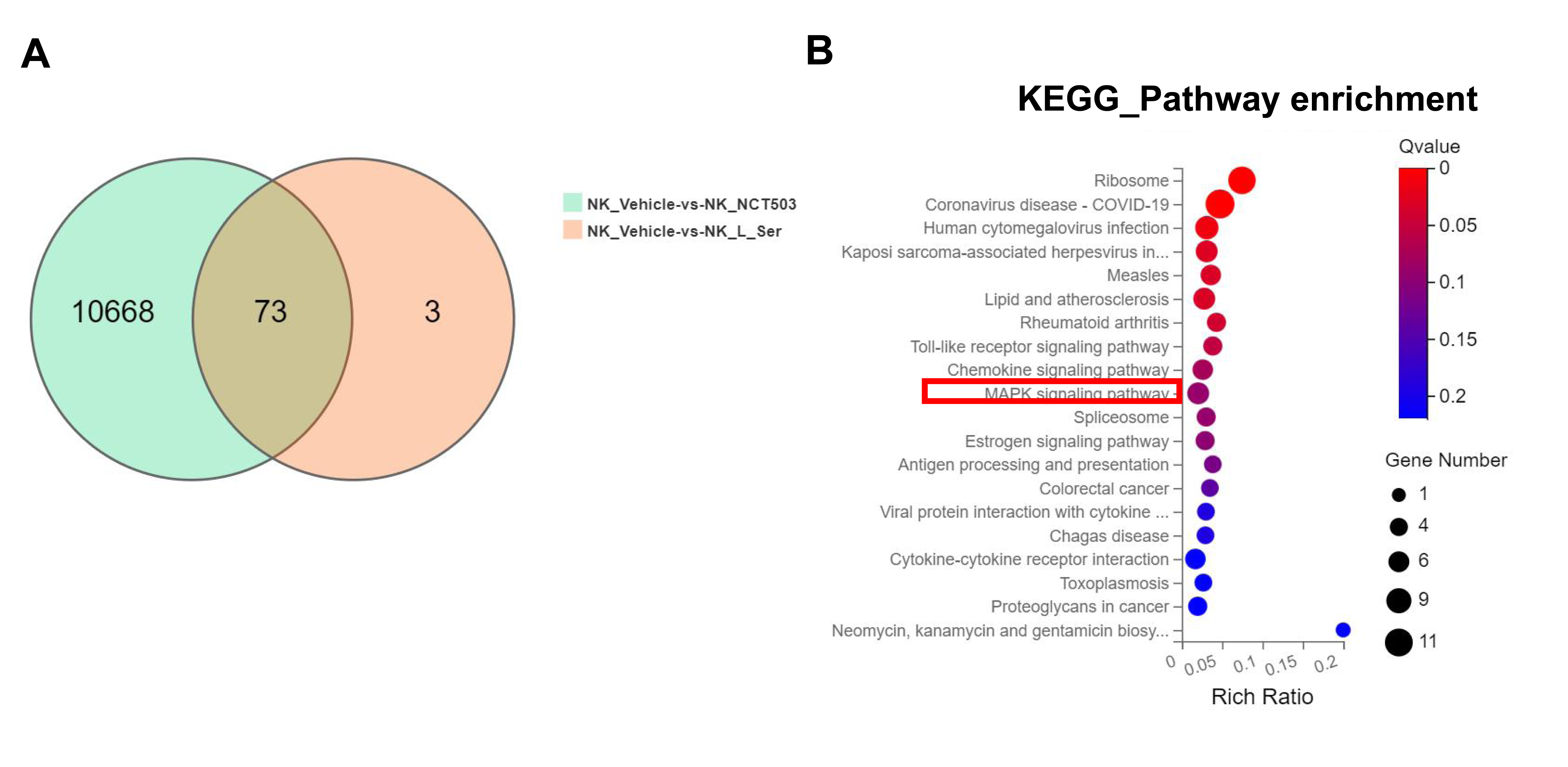


Figure S4: RNA-seq analyses of L-Serine and NCT503 on the gene expression profile. A. Venn diagram of L-Serine and NCT503 differential genes; B. Enrichment map of KEGG functional pathway of differential genes in NCT503 treatment group.


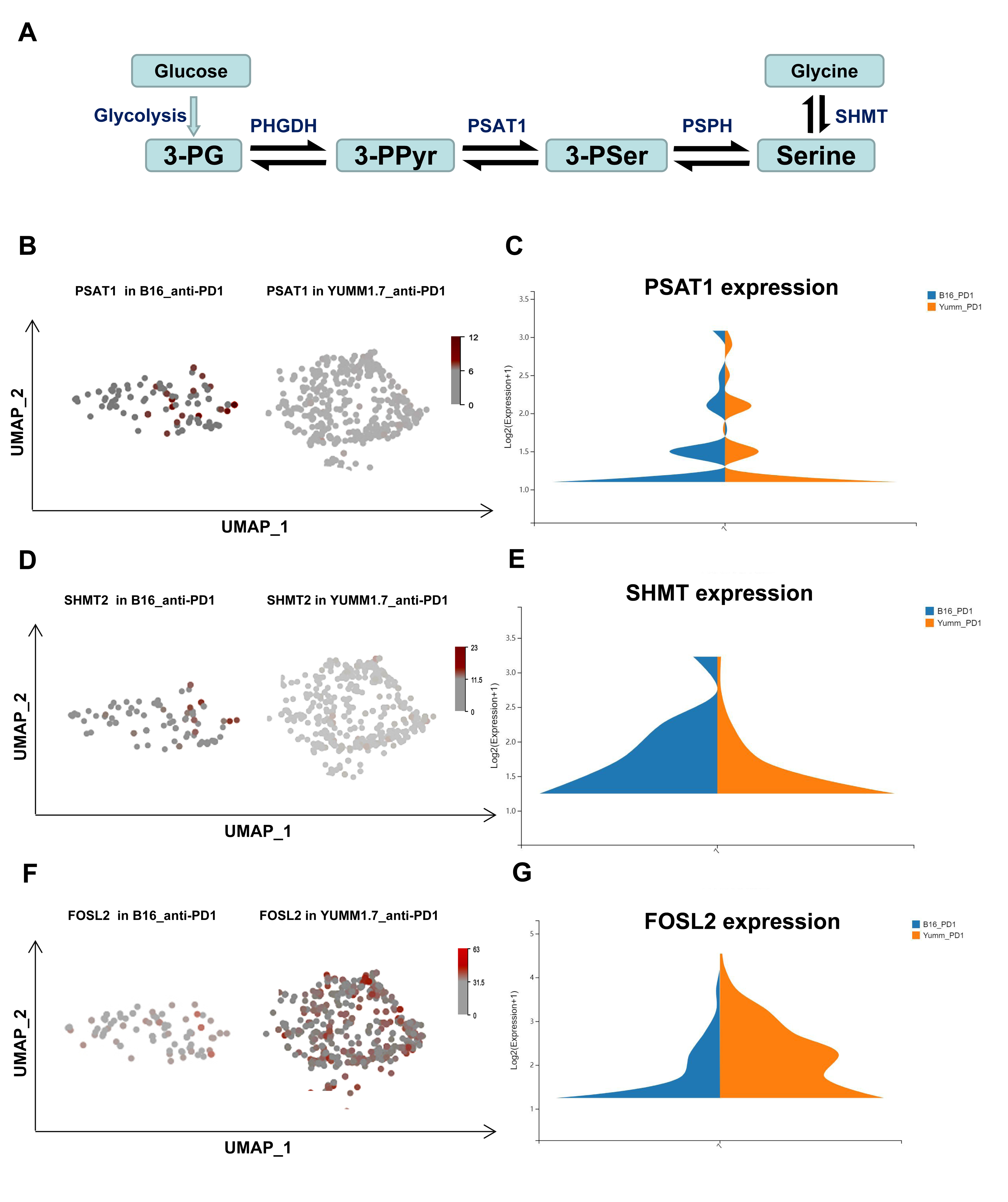


Figure S5: L-Serine metabolizing enzymes in NK cells were less expressed in CR group than in the PR group. A. Schematic diagram of L-Serine metabolic pathway; B-C. Single-cell sequencing results shown in PSAT1 expression for NK cells; D-E. Single-cell sequencing results shown in SHMT expression for NK cells; F-G. Single-cell sequencing results shown in FOSL2 expression for NK cells.


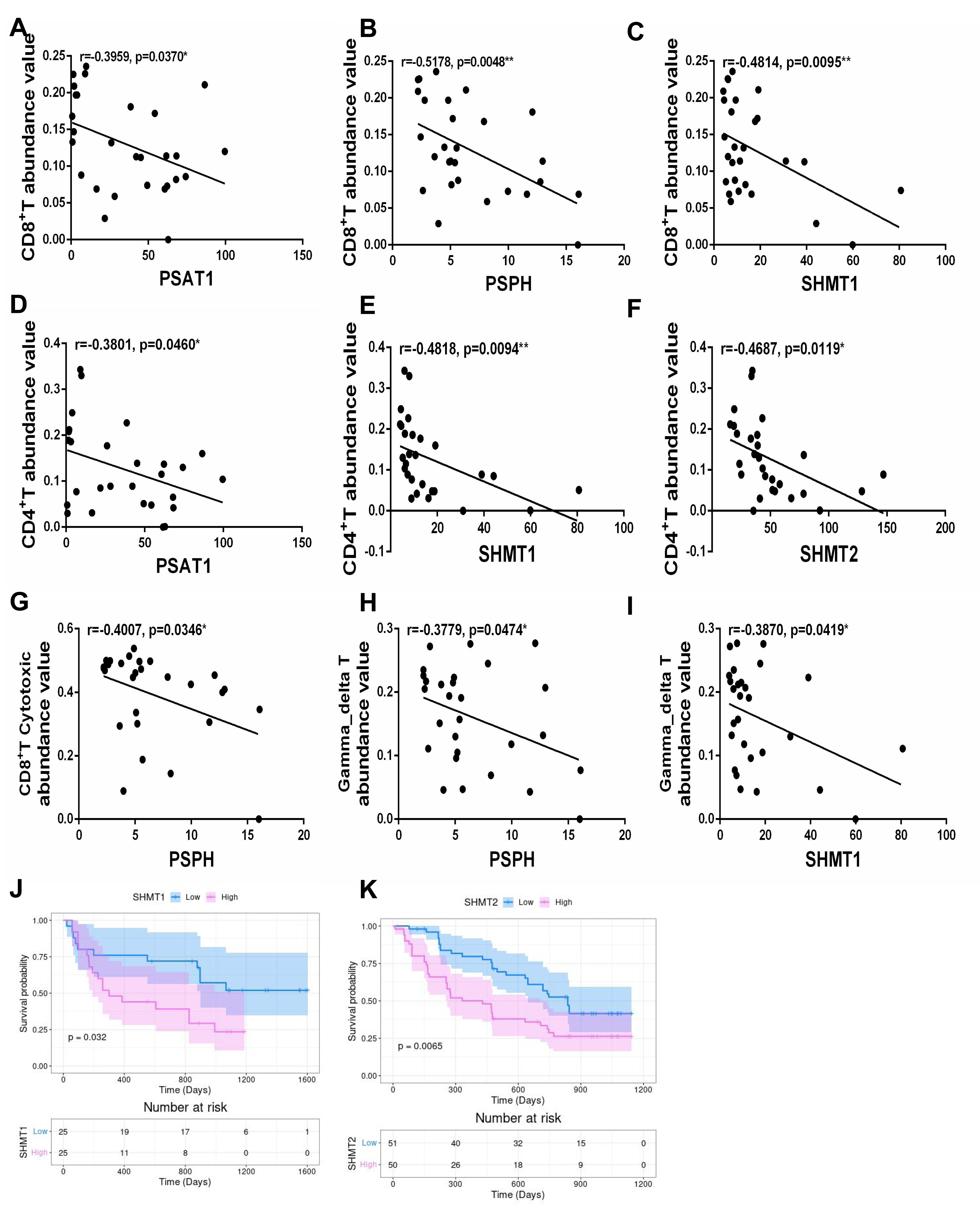


Figure S6: Correlation of L-Serine metabolic pathway genes with PD1 treatment efficacy. A-C. Correlation analysis of the serine-metabolizing enzymes PSAT1 (A), PSPH (B) and SHMT1 (C) with CD8^+^ T-cell abundance in anti-PD1-treated patients. D-F. Correlation of PSAT1 (D), SHMT1 (E) and SHMT2 (F) with CD8^+^ T-cells in PD1-treated patients; G. Correlation of L-Serine metabolizing enzyme PSPH with CD8+ cytotoxic T-cell abundance in PD1-treated patients; H-I. Correlation of PSPH (H) and SHMT1 (I) with gamma-delta T-cells in PD1-treated patients. J-K. Survival curves of patients with SHMT1(J) and SHMT2(K) expression in the R and NR groups. Multiple experimental data were counted and are presented according to the statistical method, and an asterisk (*) indicates p value.

**Table S1: Clinical information of the sample of 27 melanoma patients treated with ICI (cohort 1)**

| Sample name | Sampling time point | | Immunotherapeutic agent | | | Clinical benefit |  |
| --- | --- | --- | --- | --- | --- | --- | --- |
| SRR9033724 | Baseline | | PD1 | | | NR |  |
| SRR9033725 | Baseline | | PD1 | | | NR |  |
| SRR9033722 | Baseline | | CTLA4/PD1 | | | R |  |
| SRR9033723 | Baseline | | CTLA4/PD1 | | | NR |  |
| SRR9033715 | Baseline | | PD1 | | | NR |  |
| SRR9033716 | Baseline | | CTLA4/PD1 | | | NR |  |
| SRR9033718 | Baseline | | CTLA4/PD1 | | | R |  |
| SRR9033754 | Baseline | | CTLA4/PD1 | | | R |  |
| SRR9033753 | Baseline | | PD1 | | | NR |  |
| SRR9033750 | Baseline | | PD1 | | | R |  |
| SRR9033749 | | Baseline | | PD1 | R | | |
| SRR9033752 | | Baseline | | PD1 | R | | |
| SRR9033751 | | Baseline | | PD1 | R | | |
| SRR9033746 | | Baseline | | CTLA4/PD1 | NR | | |
| SRR9033745 | | Baseline | | CTLA4/PD1 | R | | |
| SRR9033748 | | Baseline | | CTLA4/PD1 | NR | | |
| SRR9033747 | | Baseline | | PD1 | R | | |
| SRR9033720 | | Baseline | | CTLA4/PD1 | R | | |
| SRR9033721 | | Baseline | | PD1 | R | | |
| SRR9033732 | | Baseline | | PD1 | NR | | |
| SRR9033727 | Baseline | | PD1 | | | R |  |
| SRR9033743 | Baseline | | CTLA4/PD1 | | | R |  |
| SRR9033742 | Baseline | | CTLA4/PD1 | | | R |  |
| SRR9033737 | Baseline | | PD1 | | | NR |  |
| SRR9033760 | Baseline | | CTLA4 | | | NR |  |
| SRR9033719 | Baseline | | PD1 | | | NR |  |
| SRR9033738 | Baseline | | CTLA4/PD1 | | | R |  |

**Table S2: Clinical information of the sample of 25 melanoma patients treated with PD1 mAb (cohort 2**)

| Sample name | Sampling time point | Immunotherapeutic agent | Clinical benefit |
| --- | --- | --- | --- |
| ERR2162200 | Baseline | PD1 | NR |
| ERR2162201 | Baseline | PD1 | R |
| ERR2162202 | Baseline | PD1 | NR |
| ERR2162203 | Baseline | PD1 | R |
| ERR2162204 | Baseline | PD1 | NR |
| ERR2162205 | Baseline | PD1 | R |
| ERR2162206 | Baseline | PD1 | R |
| ERR2162207 | Baseline | PD1 | NR |
| ERR2162208 | Baseline | PD1 | NR |
| ERR2162209 | Baseline | PD1 | NR |
| ERR2162210 | Baseline | PD1 | NR |
| ERR2162211 | Baseline | PD1 | R |
| ERR2162212 | Baseline | PD1 | R |
| ERR2162213 | Baseline | PD1 | NR |
| ERR2162214 | Baseline | PD1 | R |
| ERR2162215 | Baseline | PD1 | NR |
| ERR2162216 | Baseline | PD1 | R |
| ERR2162217 | Baseline | PD1 | R |
| ERR2162219 | Baseline | PD1 | R |
| ERR2162220 | Baseline | PD1 | R |
| ERR2162221 | Baseline | PD1 | R |
| ERR2162222 | Baseline | PD1 | R |
| ERR2162223 | Baseline | PD1 | NR |
| ERR2162224 | Baseline | PD1 | R |
| ERR2162218 | Baseline | PD1 | NR |

**Table S3: The primers used in the q**PCR reaction and annealing

| Gene name | Sequence (5'to3') | Direction |
| --- | --- | --- |
| Eur | GTCGTACTAGAGTGTCGGA | Forward |
| Eur | CCTCAGCGTCAGTTATCG | Reverse |
| 7F | CAGAGTTTGATCCTGGCT | Forward |
| 1640R | AGGAGGTGATCCAGCCGCA | Reverse |
| PFN2(Human) | TCAGAGCATTACGCCAATAGAA | Forward |
| PFN2 (Human) | CTGATCACTGAGCATTTCTTCG | Reverse |
| PFN2 (Mouse) | CTACGTGGATAACCTGATGTGC | Forward |
| PFN2 (Mouse) | TCGCAGTAGCCGACAATGG | Reverse |
| IL13(Human) | CATGTCCGAGACACCAAAATC | Forward |
| IL13 (Human) | CCCTCGCGAAAAAGTTTCTTTA | Reverse |
| CCL2 (Mouse) | TTAAAAACCTGGATCGGAACCAA | Forward |
| CCL2 (Mouse) | GCATTAGCTTCAGATTTACGGGT | Reverse |
| CCL3(Mouse) | CTGAGATTAGAGGCAGCAAGGAACC | Forward |
| CCL3(Mouse) | TGAAGAGTCCCTGGATGTGGCTAC | Reverse |
| IL13 (Mouse) | CCTGGCTCTTGCTTGCCTT | Forward |
| IL13 (Mouse) | GGTCTTGTGTGATGTTGCTCA | Reverse |
| PFN1(Mouse) | AAAGACCGGTCAAGTTTTTACG | Forward |
| PFN1(Mouse) | CTTGGTGACAGTGACATTGAAG | Reverse |
| IL21(Mouse) | GGACCCTTGTCTGTCTGGTAG | Forward |
| IL21 (Mouse) | TGTGGAGCTGATAGAAGTTCAGG | Reverse |
| CCL2(Human) | ACCAGCAGCAAGTGTCCCAAAG | Forward |
| CCL2(Human) | TTTGCTTGTCCAGGTGGTCCATG | Reverse |
| IL21(Human) | AGCTTTTTCCTGCTTTCAGAAG | Forward |
| IL21(Human) | GTGGTTTCCTCTTCAGCTTTTT | Reverse |
| FOS(Human) | TTACTACCACTCACCCGCAGACTC | Forward |
| FOS(Human) | GGGAATGAAGTTGGCACTGGAGAC | Reverse |
| FOSL2(Human) | ACCTCCACACCTGCTGTCACTC | Forward |
| FOSL2(Human) | CTGCTACTGCTTCTGCGGTGAG | Reverse |
| Actb (Mouse) | GGGAAATCGTGCGTGAC | Forward |
| Actb (Mouse) | AGGCTGGAAAAGAGCCT | Reverse |
| Actb (Human) | CTACCTCATGAAGATCCTCACCGA | Forward |
| Actb (Human) | TTCTCCTTAATGTCACGCACGATT | Reverse |
